# '*There is no point giving cash to women who don't spend it the way they are told to spend it*' – Exploring women's agency over cash in a combined participatory women's groups and cash transfer programme to improve low birthweight in rural Nepal

**DOI:** 10.1016/j.socscimed.2018.12.005

**Published:** 2019-01

**Authors:** Lu Gram, Jolene Skordis-Worrall, Naomi Saville, Dharma S. Manandhar, Neha Sharma, Joanna Morrison

**Affiliations:** a Institute for Global Health, University College London, 30 Guilford Street, WC1N 1EH, United Kingdom; b Mother Infant Research Activities, YB Bhawan, Thapathali, Kathmandu, 921, Nepal

**Keywords:** Nepal, Agency, Gender, Cash transfers, Participatory learning and action, Process evaluation, Maternal health, Newborn health

## Abstract

Cash transfer programmes form an integral part of nutrition, health, and social protection policies worldwide, but the mechanisms through which they achieve their health and nutritional impacts are incompletely understood. We present results from a process evaluation of a combined participatory women's groups and cash transfer programme to improve low birth weight in rural Nepal. We explored the ways in which context, implementation, and mechanism of the intervention affected beneficiary women's agency over cash transfers. Informed by a grounded theory framework, we conducted and analysed semi-structured interviews with 22 beneficiary women, 15 of their mothers-in-law, 3 of their elder sisters-in-law and 20 husbands, as well as a focus group discussion with 7 supervisors of the women's group intervention. Our study reveals how women's group facilitators, their supervisors and community members developed a shared dynamic around persuading and compelling recipients of unconditional cash transfers into spending them according to criteria developed by the group. We found these dynamics effectively constituted ‘soft conditions’ on beneficiary spending which restricted women's ability to make decisions over their cash transfers, but also increased their likelihood of spending them on their own pregnancy. Our findings demonstrate the importance of understanding how programmes are implemented and responded to in order to understand their implications for beneficiary agency and empowerment.

## Highlights

• We studied a combined participatory women's group and cash transfer intervention.
• Group facilitators and community members created social pressure over transfer use.
• These social pressures effectively constituted ‘soft conditions’ on cash transfer use.
• Participatory women's groups can promote beneficiary spending on health & nutrition.

## 1 Introduction

Cash transfer programmes have become a ubiquitous feature of nutrition, health, and social protection policy worldwide (Honorati et al., 2016). Cash transfers have been found to have a variety of beneficial outcomes, including reduced poverty, increased health service utilisation, and raised school attendance (Bastagli et al., 2016). Within nutrition, studies to date have found greater impacts of food transfers than cash transfers on anthropometric outcomes (Ahmed et al., 2009; Hidrobo et al., 2014; Saville et al., 2018), but cash transfers are often preferred in situations where logistical and administrative difficulties with procuring, storing and transporting food are significant (Ahmed et al., 2009). Thus, finding ways to increase the impact of cash transfers is desirable. Nevertheless, the pathways through which they achieve health and nutritional impacts remain incompletely understood (Gaarder et al., 2010).

Existing theories of change (Gaarder et al., 2010; Leroy et al., 2009; Groot et al., 2017) emphasise beneficiary spending of cash transfers as a mediator of nutrition and health impact. Cash transfers may enable beneficiaries to pay for higher quantity and quality of food as well as curative and preventative health visits, transportation to health facilities, and medical supplies. However, beneficiary spending of cash transfers does not happen in a vacuum, but is influenced by individual beliefs, attitudes and relationships to other household members (Zembe-Mkabile et al., 2018; Scott et al., 2017; Tonguet-Papucci et al., 2017; Junior et al., 2016). These influences are frequently the target of complementary behaviour change components (Molyneux and Thomson, 2011; Attanasio et al., 2009; Barca et al., 2015; Robertson et al., 2014; Adato and Hoddinott, 2010), but their mechanism of operation remains little explored.

A notable exception is the literature on ‘labelling’ effects, which studies the effect of providing clear messages to beneficiaries about the purpose of cash transfers (Benhassine et al., 2015; Pace et al., 2016, 2018; Kilburn et al., 2017). Households often do not treat all income as fungible, but over-spend cash labelled with a specific purpose on their perceived purpose as compared to a generic income effect (Islam and Hoddinott, 2009; Thaler, 1999; Jacoby, 2002; Beatty et al., 2014). A cluster randomised controlled trial in Morocco compared conditional cash transfers – where beneficiaries had to send their children to school to continue to receive them – with labelled cash transfers – where beneficiaries received flyers recommending spending on schooling (Benhassine et al., 2015). The researchers found both interventions increased schooling outcomes to an equal extent. They hypothesised that their labelling component may have encouraged beneficiary households to spend cash on schooling by highlighting its value.

Nonetheless, labelling alone may provide an insufficient nudge to promote healthy behaviour change in contexts where gender and age hierarchies restrict young women's access to food and cash (Harris-Fry et al., 2017). Qualitative studies in Nepal, India and Bangladesh have found discriminatory food allocation and low decision-making power prevent high dietary intake during pregnancy (Shannon et al., 2008; Zaidi, 1996; Morrison et al., 2018). Studies of financial power in South Asia have also found significant risks of economic violence and exploitation by other household members for young women who are rarely in a position to openly demand access to cash (Chowbey, 2017; Kabeer, 1997; Singh and Bhandari, 2012; Gram et al., 2018a). In such contexts, simply labelling cash transfers as belonging to beneficiary women may be insufficient to protect their *agency* over cash transfers – i.e. their freedom to spend their transfers without interference from other household members.

Our present study describes and analyses the process through which a complementary participatory women's group intervention may have affected beneficiary agency over cash transfers in a combined women's group and cash transfer intervention to improve birth weight in rural Nepal (Saville et al., 2018). The cash transfers were ‘unconditional’ in that there were no formal requirements associated with receiving them, but ‘labelled’ in that facilitators clearly communicated their purpose in group meetings as supporting pregnant women's health and nutrition. However, the women's groups were also designed to go beyond labelling by mobilising collective action from community and group members to support pregnant women achieve their nutrition and health goals. A cluster-randomised controlled trial did not find a statistically significant impact of cash transfers and women's groups on birthweight in the first 72 h, but inclusion of a larger sample of weights up to 10 days after birth did demonstrate a statistically significant 69 g increase compared with control (Saville et al., 2018). A dietary sub-study also found substantial improvements in pregnant women's dietary diversity, micronutrient adequacy, and consumption of dairy foods (Harris-Fry et al., 2018a).

A key initial hypothesis was that participatory women's groups could enhance pregnant women's agency over their cash transfers. Group members might raise awareness about the importance of caring for women's health or support women to become more confident in demanding control over the cash transfer from other household members. To explore this hypothesis, we conducted a qualitative process evaluation (Moore et al., 2015) informed by grounded theory (Corbin and Strauss, 2008). Our main research question was: how did the context, implementation and mechanism of our participatory women's group intervention affect beneficiaries' agency over cash transfers?

## 2 Study setting

### 2.1 Geographical setting

Our study took place in the Dhanusha and Mahottari districts in the Plains of Nepal, bordering Bihar state in India. 21% of households are classified as ‘severely food insecure’ and levels of undernutrition in women and girls are high (Ministry of Health and Population (MOHP) [Nepal], New ERA, & ICF International Inc., 2012). Male foreign labour migration is widespread with top destinations being India, Qatar, Kuwait, United Arab Emirates and Malaysia (Ministry of Labour and Migration, 2014). The local language and culture is Maithili. Most married women live with their husband's families in extended family households. Newly married women's mobility outside the home is restricted by social norms of female propriety and modesty (Davis, 2009); they are almost always escorted by their husband or an elder male/female in-law when moving around outside the home (Davis, 2009). Purchases of goods from the market are almost entirely mediated by male family members or elder female in-laws (Gram et al., 2018a). Inside the home, newly married women are expected to defer to their husband or elder family members in decision-making (Davis, 2009). Usually, a senior household member, such as the mother-in-law, physically receives and manages the majority of household income on behalf of other household members, including her son (Gram et al., 2018a). Women living in nuclear families have much greater decision-making power and mobility as the *de facto* oldest woman of their household (Gram et al., 2018a).

### 2.2 Trial setting

Our study was embedded in a cluster-randomised controlled trial comparing the impact of three interventions on birth weight relative to control (Saville et al., 2016): 1) women's groups practising participatory learning and action 2) women's groups combined with monthly unconditional food transfers to pregnant women 3) women's groups combined with monthly unconditional cash transfers to pregnant women. The unit of randomisation was the Village Development Committee (VDC), a sub-district level geographical area with approximate populations of 6000 each. 80 VDCs were randomised to one of the four above treatments resulting in 20 VDCs per trial arm. Trial interventions ran from 2013 until 2015. Mother Infant Research Activities (MIRA) implemented trial interventions in collaboration with University College London. The full details of trial design and eventual outcomes have been reported elsewhere (Saville et al., 2016; Saville et al., 2018).

### 2.3 Design of the combined women's groups and cash transfer intervention

Trained facilitators convened and held monthly group meetings using a pictorial manual. Any women could come to group meetings, and most pregnant women attended with their mother-in-law. Facilitators led group members through a cycle of problem identification and prioritisation, strategy formulation and implementation, and participatory evaluation to address local health problems. Facilitators discussed a wide variety of health and nutrition-related topics using a pictorial manual, including micronutrient intake, iron supplementation, deworming medicine, hygiene and sanitation, family support and rest, antenatal care visits, birth preparedness and newborn care. Facilitators emphasised the importance of ensuring dietary diversity and particularly recommended spending the cash transfers on micronutrient-rich foods like vitamin A-rich fruits and vegetables (such as mangoes and green leafy vegetables), and animal source foods (dairy, eggs, fish, and meat), supplemented with home-grown fruits and vegetables.

All pregnant women in a village were eligible for monthly transfers of NPR 750 once their pregnancy was detected and verified and a photo ID card had been issued (Saville et al., 2016). The NPR 750 cash transfer comprised 32% of the total monthly cost of a nutritionally adequate food basket for a pregnant woman in 2011. Cash transfers were distributed at the end of women's group meetings. Each woman was asked how they had spent their transfers in the previous month before being given their next transfer. Registered women were not required to attend entire meetings to receive their transfer but could pick it up at the end of a meeting or from a group facilitator the next day. Registered women who were physically unable to attend received their transfers at home. A post-distribution monitoring team from Save the Children performed home visits every month in 5% of beneficiary households asking household members questions on how they had spent the transfer. Women ceased to be eligible for further transfers after the end of their pregnancy.

## 3 Methods

### 3.1 Study design

We conducted a qualitative process evaluation (Moore et al., 2015) informed by grounded theory (Corbin and Strauss, 2008). We iterated between data collection and data analysis and sampled towards saturation to generate a theory of cash transfer use (Corbin and Strauss, 2008; Corbin and Strauss, 2008). We chose grounded theory to maximise our chances of inductively developing a nuanced theory with a coherent logic where all the theoretical categories are connected to one another and no major explanatory gaps are left.

### 3.2 Data collection

To collect data on intervention context, implementation and mechanisms of effect, we conducted one focus group discussion with 7 women's group supervisors who attended the central office for a meeting. Across the women's group and cash arm of the trial, 16 supervisors coached facilitators at monthly meetings, in which they actively participated themselves to support group facilitators. We took informed consent from all seven supervisors; none refused.

We also conducted semi-structured interviews with 22 beneficiary women and their guardians, whether mothers-in-law (15) or elder sisters-in-law (3) (Table 1). All interviewees gave informed consent. None of the beneficiary women refused consent, but three guardians refused consent because they did not have time. In these cases, we still interviewed the beneficiary woman alone. The recording of one mother-in-law was lost due to equipment failure. We purposively sampled to obtain variation in geographical area, caste, socio-economic status, education, nuclear versus joint household, and husband's migrant status. All the sampled beneficiaries had received at least three cash transfers. Due to low levels of ethnic variability in our geographical context, we did not sample based on ethnicity. We did not explicitly sample guardians based on any criteria, but instead interviewed any consenting guardian of a sampled daughter-in-law. Locally resident MIRA staff identified women matching our sampling criteria and invited them to participate.

**Table 1:** Female respondent characteristics.

Household characteristics						Mother-in-law/Elder sister-in-law to beneficiary		Beneficiary woman		
Serial no.	Area	Caste	SES	Nuclear/Joint family	Husband working abroad?	Family relation	Grades passed	Age	Grades passed	Pregnant?
1	Sahku	Dalit	Middle	Joint	No	Mother-in-law	0	20	7	No, baby 5 months
2	Sahku	Dalit	Low	Nuclear	Yes, ME	Mother-in-law	0	25	0	No, baby 7 months
3	Sahku	Brahmin	High	Joint	No	Sister-in-law	7	21	8	No, baby 6 months
4	Sahku	Brahmin	High	Joint	No	Mother-in-law	0	21	8	No, baby 4 months
5	Sahku	Middle Madhesi	Low	Nuclear	No	Mother-in-law	0	21	7	No, baby 1.5 months
6	Sahku	Middle Madhesi	Middle	Joint	No	Mother-in-law	0	21	5	Yes, 7 months
7	Sahku	Middle Madhesi	High	Joint	Yes, ME	Mother-in-law	0	24	8	No, baby 5 months
8	Sahku	Brahmin	High	Joint	No	Mother-in-law	7	20	12	No
9	Sahku	Dalit	Low	Joint	No	Mother-in-law	0	30	0	No, baby 5 months
10	Sahku	Middle Madhesi	High	Nuclear	No	MIL refused consent		21	0	Yes, 8 months
1	Kote	Dalit	Middle	Joint	No	Mother-in-law	0	20	14	Yes, 8 months
2	Kote	Dalit	Low	Nuclear	No	Equipment failure		25	5	No, baby 5 months
3	Kote	Dalit	Low	Joint	Yes, ME	Sister-in-law	0	18	6	No, baby 1 month
4	Kote	Dalit	Low	Joint	Yes	Mother-in-law	0	25	8	No
5	Kote	Dalit	Middle	Nuclear	Yes, ME	Mother-in-law	0	25	0	Yes, 7 months
6	Kote	Dalit	Middle	Joint	No	Mother-in-law	0	20	0	No, baby 1 month
7	Kote	Dalit	Middle	Joint	No	Mother-in-law	0	22	12	No, baby 7 months
8	Kote	Middle Madhesi	Low	Joint	Deceased	Mother-in-law	0	20	3	No, baby 2 months
1	Jamal	Dalit	Middle	Joint	Yes	MIL refused consent	20	0	No, baby 4 months	
2	Jamal	Middle Madhesi	High	Joint	Yes, Malaysia	Mother-in-law	0	25	8	No, baby 6 months
3	Jamal	Dalit	Low	Nuclear	No	Sister-in-law	0	25	8	No, baby 3 months
4	Jamal	Dalit	Low	Joint	Yes, Delhi	MIL refused consent	17	5	No	

ME = Middle East. Socioeconomic status was appraised by local informants and adjusted based on interview data in case of discrepancies. Destination country of migration was not reported for respondent 4, Kote and 1, Jamal. Ages for mothers-in-law/elder sisters-in-law not provided, as mothers-in-law had difficulties reporting their age exactly, although it ranged from 50 to 80 years. All respondents were of Hindu faith. For daughters-in-law 8 from Sahku, 4 from Kote and 4 Jamal, the age of the baby was not reported.

Pilot interviews indicated that mothers-in-law were reluctant to let us interview their beneficiary daughter-in-law in private, while daughters-in-law felt intimidated by the prospect of discussing household matters in front of their mother-in-law. We therefore conducted concurrent but separate interviews with daughters-in-law and their guardian, in two separate locations around the household. While the daughter-in-law was interviewed in her room, the guardian was simultaneously interviewed in an outside location, such as under a tree or in a nearby field. This ensured a high degree of privacy for the daughter-in-law, as male household members were at work and other female household members may have presumed our interview with the mother-in-law was more significant and of greater interest. A similar strategy has been used during interviews in high-income settings to separate married women from their husbands (Hertz, 1995).

We interviewed 20 husbands from households that participated in the trial (Table 2). Husbands were sampled based on variation in geographical area, caste, socio-economic status, education, and nuclear versus joint household. We used the surveillance system of the trial to identify them. We did not revisit the original households where we had interviewed female household members because there was a delay of three months between data collection with men and women due to funding constraints and we sought to minimise time burdens on households. Therefore, husbands were unrelated to our earlier female respondents. All husbands gave informed consent. It was often difficult to find a suitable time to interview husbands due to their need to leave for work early in the morning and spend time with the family in the evening. Five refused to participate because they did not have time. One expressed offence at being asked questions that ‘should be asked of women only’. All husbands were interviewed alone under a tree in an open field.

**Table 2:** Male respondent characteristics.

Serial no.	Area	Caste	SES	Nuclear/joint household	Age	Grades passed	Interview taken?	Wife pregnant?
1	Dahak	Middle Madhesi	II	Nuclear	40	0	Yes	No, baby 1 month old
2	Dahak	Middle Madhesi	IV	Joint	30	9	No, consent refusal	No, baby 18 days' old
3	Dahak	Middle Madhesi	IV	Nuclear	26	4	Yes	No, baby 17 days' old
4	Dahak	Middle Madhesi	V	Joint	28	8	Yes	No, baby 2 months old
5	Dahak	Dalit	I	Nuclear	30	0	No, consent refusal	No, baby 1 month old
6	Dahak	Dalit	I	Joint	?	0	Yes	No, baby 6 months old
7	Dahak	Middle Madhesi	IV	Joint	22	11	Yes	No, baby 1 month old
8	Dahak	Middle Madhesi	I	Joint	23	4	Yes	No, wife had an abortion
9	Dahak	Middle Madhesi	V	Joint	28	13	No, consent refusal	Unknown
10	Dahak	Muslim	I	Joint	26	0	No, moved abroad	Unknown
11	Dahak	Dalit	I	?	?	0	No, consent refusal	Unknown
1	Lalit	Middle Madhesi	III	Joint	23	6	Yes	No, baby 14 days' old
2	Lalit	Middle Madhesi	V	Joint	38	8	Yes	No, baby 1 month old
3	Lalit	Middle Madhesi	I	Joint	32	6	Yes	No, baby 1 month old
4	Lalit	Middle Madhesi	III	Joint	31	0	Yes	Yes
5	Lalit	Middle Madhesi	II	Nuclear	29	0	Yes	Yes
6	Lalit	Middle Madhesi	IV	Nuclear	41	8	Yes	Yes
7	Lalit	Middle Madhesi	III	Joint	24	11	Yes	No, baby 1 month old
8	Lalit	Dalit	II	Joint	24	5	No, consent refusal	Unknown
9	Lalit	Middle Madhesi	V	Joint	26	10	No, moved abroad	Unknown
1	Jamal	Middle Madhesi	V	Joint	28	6	Yes	No, baby 1 month old
2	Jamal	Middle Madhesi	III	Nuclear	32	5	Yes	No, baby 1 month old
3	Jamal	Dalit	I	Joint	25	0	Yes	No, baby 1 day old
4	Jamal	Muslim	III	Joint	35	0	Yes	Yes
5	Jamal	Middle Madhesi	III	Joint	28	0	Yes	No, wife suffered miscarriage
6	Jamal	Muslim	III	Nuclear	44	0	Yes	No, baby died
7	Jamal	Middle Madhesi	V	Joint	25	7	Yes	No, baby 2 months old
8	Jamal	Muslim	I	Joint	40	0	No, consent refusal	Unknown

SES reflects socioeconomic quintiles calculated using an asset index (Filmer and Pritchett, 2001) from surveillance data, I = lowest socioeconomic status, V = highest. Husbands 6 and 11 in Dahak did not provide information on their age. Husband 11 in Dahak did not provide information on family structure.

Author NSh conducted interviews with beneficiary women, while female women's group supervisors interviewed their mothers-in-law or elder sisters-in-law. NSh also conducted the focus group discussion with women's group supervisors. A male researcher interviewed male respondents. All interviewers were native Maithili speakers. NSh and the women's group supervisors had previous experience with conducting interviews for MIRA NGO, while the male interviewer was directly trained by authors LG and JM to conduct interviews.

Data were collected from April until November 2015. All interviews were audiotaped and lasted between one and two hours. Native Maithili speakers directly transcribed and translated the data from the audio recording into written English.

### 3.3 Data analysis

For each transcript, reflective notes were written using analytical tools from grounded theory followed by open, axial and selective coding (Corbin and Strauss, 2008). Coding was carried out in MAXQDA 2018. We triangulated our findings between daughters-in-law, mothers-in-law/sisters-in-law, husbands and NGO supervisors to explore the extent that our concepts were shared across staff and household members.

Author LG performed the coding and analysis of the translated data. LG was resident in Nepal for most of the period from the 1st of April until the 1st of September 2015 and worked with the NSh and other interviewers during this period. To ensure analytic rigour, LG, NSh and other interviewers discussed findings and interpretations of the data throughout data collection. LG also listened to the audio files and consulted with interviewers and translators throughout the analysis to check understanding of transcripts and avoid cultural bias in the interpretation of the data. Senior co-authors who were permanent residents of Nepal reviewed the data (JM) and the presentation of themes and discussion (JM and NSa) to reduce bias, stimulate reflective analysis and reduce the influence of LG's position.

## 4 Results

In the following sections, we present our results using the UK Medical Research Council's process evaluation framework (Moore et al., 2015): *context* refers to the social, economic and cultural factors shaping the implementation and mechanism of an intervention; *implementation* refers to intervention delivery such as the training and performance of staff or the reach and coverage of an intervention; *mechanism* refers to participant responses and interactions with an intervention and causal mediators of intervention impact; *output* refers to beneficiary agency over cash transfers. Quotes are labelled with pseudonyms for the areas where interviews were conducted and a serial number. Quotes from the focus group discussion with women's group supervisors are marked [FGD].

### 4.1 Context

#### 4.1.1 Low perceived purchasing power of cash transfers

Across the wealth spectrum, beneficiary women stated that the transfer was a small amount of money or “*nothing*” [beneficiary 6, Kote], they would “*eat nothing*” if its size was halved [beneficiary 4, Kote], and “*nothing could be bought*” with it [beneficiary 1, Jamal]. For poorer families, the transfer was not perceived to be enough to meet their food needs, as rapid inflation during the study period decreased the purchasing power of the transfer. For better-off families, the NPR 750 [USD 6.98 in July 2016] transfer appeared an insignificant amount when compared with foreign remittance income that they often received (NPR 20,000–100,000 monthly per earner) as well as compared with costs of maternity care (NPR 50,000 for a C-section, NPR 2000–10,000 for ultra-sound scans, and NPR 3500–8000 for an antenatal care visit).

The low perceived purchasing power of the cash transfer was exacerbated by a widespread belief by households that the group facilitators had explicitly endorsed feeding apples and pomegranates, some of the most expensive non-meat foods available, to the pregnant woman “*in maximum quantity*” [elder sister-in-law 3, Jamal]. One kg of apples cost NPR 100–180, while one kg of pomegranate cost NPR 200–300, thus nearly the entirety of the monthly transfer could be spent on buying 4–7 kg apples or 3–4 kg pomegranates. Compared to such prices, half a litre of milk cost only NPR 25, potatoes cost NPR 20 per kg, rice cost NPR 50 per kg, and lentils cost NPR 100 per kg. Meat and fish cost NPR 300–400 per kg and one bottle of beer cost NPR 250. Ironically, the high price of apples and pomegranates itself may have served to signal their nutritional value “*The [facilitators] had told us to eat good things and you know that good things cost a lot*” [daughter-in-law 6, Sahku]. A local belief also held that pomegranates build blood for the unborn baby due to their red colour.

The low perceived purchasing power of cash transfers created significant difficulties for group facilitators and their supervisors with communicating the value of the cash transfer.

Women's group supervisor: Those who have money, they say that NPR 750 is nothing and more should be added to it. But we told them that NPR 750 is a big amount if you know how to use it … there is no limit to the need of money … everyone has thousands and thousands of rupees, but they do not spend it on food. They spend it on building big houses if they have small ones, or on buying fields … [But] even they themselves cannot afford to donate NPR 750 to someone just like that. No. What those NPR 750 rupees can and cannot buy for one single person, they should be taught about that.

[FGD]

### 4.2 Implementation

#### 4.2.1 Emergent negotiations over cash transfer use

Originally, group facilitators were only meant to provide broad recommendations to spend the transfer on micronutrient-rich foods for pregnant women and reflect on ways to enhance women's agency over cash transfers in an open-ended manner with group members, if necessary. However, their difficulties with communicating the value of the cash transfer catalysed fears that families might confiscate it or that beneficiaries might ‘waste’ [FGD] it on non-health-related purposes and forego an opportunity to improve birth weight. To prevent this, group facilitators and their supervisors decided to create budgeting recommendations and ‘rules’ [FGD].

First, facilitators and their supervisors created a recommendation advising families to spend approximately NPR 25 a day over one month on milk. Facilitators used a picture of a calendar from their manual to make this point clear. However, poor families complained that they could not justify spending the entire cash transfer on only milk, when they could not afford staple foods, while wealthier families wanted to make bulk purchases of more expensive fruits; they may have had plenty of access to own-produced milk. In response to such requests, the facilitators created an additional ‘rule’ [FGD] that wealthier participants should plan ahead so that expenditures averaged out to NPR 25 a day, while poorer participants should spend half the money on milk and half on staple foods. However, families also wanted to save up their cash transfer to plan for private maternity and delivery care. Facilitators responded to such requests by creating a third recommendation advising families not to spend their cash transfer on delivery costs, but to save up money from other sources. Finally, facilitators and their supervisors felt beneficiary women might benefit from an explicit recommendation to households to let beneficiaries control the cash transfers. After asking group members if they approved of such a rule and receiving an answer in the affirmative, facilitators announced another ‘rule and regulation’ [FGD] that beneficiary women had to physically keep their cash transfers.

#### 4.2.2 Enforcing recommended usage of cash transfers

Group facilitators and their supervisors worried that simply disseminating recommendations on cash transfer use was insufficient to achieve successful outcomes [FGD]. Therefore, facilitators and their supervisors began to take more active measures to promote compliance. In early group meetings, they observed beneficiaries handing over their cash transfers to their mothers-in-law as soon as they received them. Facilitators and their supervisors decided to intervene whenever they observed them handing over their cash transfer to other family members [FGD].

To ensure households spent the transfer as recommended, some facilitators and their supervisors personally visited households suspected of not following spending guidance based on information from community members to ‘cross-examine’ [FGD] household members. Such cross-examinations were not always conducted in a supportive manner: One beneficiary narrated how a facilitator would repeatedly ask her about her spending behaviour and asked to be shown the fruit she had purchased with the cash transfer. To indicate the level of perceived scrutiny, the beneficiary woman used the expression ‘*She even knows how much salt I put in pulses while cooking!*’ [beneficiary 3, Jamal].

Social pressures also arose in the monthly women's group meetings, when facilitators and women's group members reviewed and discussed how they had spent the preceding month's cash transfers. Women's group supervisors acknowledged that these were meant to be open discussions held under the premise that women had the right to the transfer and could spend it however they liked [FGD]. However, facilitators' eagerness to encourage women to adopt the desired behaviours may have led some to apply more pressure than intended. One supervisor provided a message to her community that if they did not spend the money as instructed and wasted her time by producing unhealthy children, she would stop coming to the community to distribute cash.

Women's group supervisor: We told them that there wouldn't be any further cash transfers, if we did not come and sit with them, and we had chosen to spend so much time there so that their children could grow up healthy and sound. If the children didn't grow up healthy, then it was of no use to us to come and spend so much time. The women who were not pregnant, like the mothers-in-law, they were telling the pregnant women that if they were not willing to come to the women's group, there would be too few people in the group and we would not be coming any longer. Then no more money would be given, and they would be banished from our offices. That was why, they should spend it as instructed.

[FGD]

Another supervisor told women's group members that the trial intervention would be translated into permanent government policy if beneficiaries demonstrated the potential for the intervention to prevent low birth weight, even though no such arrangement existed between MIRA NGO and the Nepal government ‘*What is wrong with giving an example? It is only to inspire them*’ [FGD]. A third supervisor described how she ‘cross-examined’ [FGD] group members because she wanted to be sure they had not spent their cash transfer on clothes or jewellery. A fourth supervisor stated that women's group participants only ended up spending the cash transfer on food after facilitators ‘*again and again told them to spend the money on food to eat*’ [FGD]. A fifth supervisor reported how she had criticised a women's group member spending the transfer on medicines for her children instead of following their guidelines on spending [FGD].

### 4.3 Mechanism

#### 4.3.1 Community action on cash transfer use

In responding to the implementation modality adopted by facilitators and supervisors, community members began taking informal action themselves to promote perceived appropriate use of cash transfers. Beneficiaries and their household members indicated community members would informally gossip about, reprimand or castigate households perceived to waste the cash transfer. A mother-in-law stated that if her daughter-in-law was still living together with her in a joint family and she took her cash transfer from her daughter-in-law, then people might start gossiping about her in the village [mother-in-law 5, Sahku]. An elder sister-in-law noted how community members would accuse pregnant women's guardians of taking cash transfers away from them if children were found to be low birth weight by a local clinic [elder sister-in-law 3, Jamal]. Many husbands remarked the purpose of the cash transfers was well-known to the wider community and thus community members would ‘*verbally abuse*’ people known to ‘*not to spend money on what they are told to spend it on*’ [husband 3, Dahak].

Consequently, household members widely condemned spending on items unrelated to health such as cosmetics, clothes or jewellery and ubiquitously asserted that the cash transfer ought to be spent on food, both spontaneously and when prompted to discuss the purpose of the cash transfer. One husband remarked that if his wife did not spend her cash transfer on food, he would demand an account of why she had not done as instructed by the women's group facilitators [husband 2, Jamal]. Another husband commented that the cash transfer programme was a huge investment that might not be made again, if households did not spend their transfers as instructed *'there is no point giving cash to women who don't spend it the way they are told to spend it'* [husband 3, Lalit]. A third husband stated that households were accountable to the women's groups for their use of the cash transfer, just like local officials are accountable to the community for their use of government budgets:

Husband: If local government officers are provided a budget to build a school, but instead of that spend the money on building houses for themselves then it is a bad thing … It is the duty of the women's groups to find out if people have actually spent the money as told.

[husband 1, Lalit]

As such, supervisors widely believed beneficiary households felt the cash transfer was labelled with a health and nutrition-related purpose “*We give them the money to buy food, thus they feel that they have to buy food with it*” [FGD]. Supervisors thought beneficiaries would feel awkward and embarrassed if it was revealed that they had spent the cash differently from its stated purpose and even thought women's own family members might inform the local women's group about such behaviour [FGD]. To describe women's fear of both personal and social feelings of guilt and shame, supervisors cited the Nepali proverb ‘*It is not the tiger of the forest that eats you alive, but the tiger of your own mind*’/*ban ko baghle nakhaeko, man ko baghle khaeko*.

#### 4.3.2 Perceived need for compliance with NGO

The many social pressures from both their community and MIRA NGO affected beneficiary women's interpretation of the meaning of their cash transfer. Beneficiary women sometimes denied making their own decisions on cash transfer use. Instead, they stated that NGO staff had taken this decision for them.

Interviewer: Who took decisions regarding use of the cash transfer in the household?

Beneficiary woman: The women's group facilitator took decisions about this.

Interviewer: Okay. But when you received the cash transfers then who took decisions regarding the use of that money?

Beneficiary woman: That decision was done by that Sir who had asked me whether we spend that money on food for ourselves or whether we give it to our family members.

[beneficiary 9, Sahku]

When asked if they had saved the cash transfer or spent it on non-food items, beneficiary women usually replied with a question about how this was possible, when they had not been told to spend their cash transfer in this way.

Interviewer: Now tell me sister, were you free to spend the cash transfer on whatever you liked to spend it on?

Beneficiary woman: No, how could I spend it on whatever I liked to spend it on?

Interviewer: Why not?

Beneficiary woman: Since it was given to me to spend exclusively on food then how could I spend it on other things?

[beneficiary 10, Sahku]

One elder sister-in-law even seemed confused about the idea that there existed women who did not spend their cash transfer on food ‘*If they have not spent the cash transfer on that, then what did they spend their cash on?*’ [elder sister-in-law 3, Jamal]. Mothers-in-law and elder sisters-in-law also widely explained that their daughters-in-law had not saved any of the cash transfer as they had not been told to do so by the NGO.

Interviewer: Did [your daughter-in-law] ever save any of the cash transfer?

Mother-in-law: She has not saved any of the cash transfer, because she was not told to do so? She was given the money to spend on fruits and vegetables. If she had been told to save, then she might save.

[mother-in-law 2, Sahku]

Women's group supervisors believed beneficiaries feared they might stop receiving the cash transfer if they were found not to have spent it on food ‘*[They think] the office will catch them*’ [FGD]. However, only one respondent, a husband, explicitly stated a belief that such a thing could happen [husband, 4 Jamal]. Another husband remarked, since community members had never observed households lose their cash transfer over how they spent it, most did not worry overly about losing their cash transfer in this way [husband 7, Dahak]. Many respondents also thought it would be challenging for our NGO to monitor how people privately spent their money, particularly as the cash received by the NGO was sometimes physically mixed in with cash from other sources by the beneficiaries. However, a few respondents indicated a belief the NGO could find out if women had eaten the food they were supposed to eat by recording and cross-checking the answers of different household members [husband 7, Jamal] or measuring the weight of the baby that was eventually born [husband 8, Dahak; elder sister-in-law 3, Jamal]. Supervisors also reported receiving news from community informants about households' use of cash transfers [FGD].

### 4.4 Output

#### 4.4.1 Beneficiary agency over cash transfers

Despite group facilitators and their supervisors' attempts to explicitly intervene when beneficiary women handed over their cash transfers to their mother-in-law, many beneficiaries simply waited until they got home before they offered it to their mother-in-law. Such beneficiaries thought it inconceivable that they could openly refuse the authority of their mother-in-law by keeping their cash transfer without even attempting to offer it to their guardian. However, in almost all cases, both daughter-in-law and mother-in-law independently confirmed that the mother-in-law had refused to accept the cash and instead insisted that the daughter-in-law should spend it on whatever she wanted to spend it on – as long it was recommended by our NGO. When asked why they had not accepted the transfer, mothers-in-law replied with a mixture of reasons: that it belonged to the beneficiary, that it was for her benefit, that they were instructed to do so, and that the transfer was of a negligible size anyway.

Such beneficiary households overwhelmingly stated that they spent their cash transfer on dairy and fruits for the pregnant woman, suggesting women's group facilitators and their supervisors largely succeeded in their attempts to ensure beneficiaries spent their cash transfers in line with their recommendations. Indeed, by far the most commonly reported pattern of expenditure was half a litre of cow milk daily at NPR 25, exactly as recommended by group facilitators, while the most commonly reported non-dairy products were apples and pomegranates. Other fruits and green vegetables were occasionally mentioned. Beneficiary women almost always relied on husbands or mothers-in-law to physically spend the transfer for them when they needed to purchase fruit due to female seclusion norms preventing young, married women from entering the marketplace.

It was clear that beneficiaries were only allowed to make ‘their own’ spending decisions concerning cash transfers, but not generic cash. When asked if their daughter-in-law was allowed to have cash savings in general, many mothers-in-law baulked at the suggestion and affirmed they as mothers-in-law were entitled to manage household income as a senior household member. When asked if their receipt of the cash transfers had led them to experience greater household decision-making power, beneficiaries usually answered with a degree of amusement: “*Such a small amount of money won't grant anyone any kind of power!*” [beneficiary 6, Kote]. Beneficiaries similarly denied experiencing greater household status after attending group meetings.

Husbands living in extended families were not offered the cash transfer, as the household hierarchy dictated deferring to in-laws before the husband in financial matters. In nuclear families, beneficiaries managed day-to-day financial transactions as the *de facto* oldest woman of their household; these reported spending their transfer on fruits and dairy. Only in one instance did a beneficiary ‘let’ her husband make decisions on the use of the cash transfer, as he insisted spending it on apples and pomegranates for her anyway [beneficiary 5, Sahku]. When asked why they had not taken the cash transfer, husbands also replied that the cash transfer was too small for them to care about and it was meant for their wife's health.

None of our respondents reported conflicts with husbands or mothers-in-law, but two beneficiaries reported conflicts with their elder sisters-in-law [beneficiary 3, Jamal; beneficiary 7, Kote]. In one instance, a beneficiary secretly spent the cash transfer food on fruits for herself with the help of her husband, as her elder sister-in-law wanted to confiscate the transfer [beneficiary 3, Jamal]. In another instance, physical violence erupted between the beneficiary and her elder sister-in-law, when the beneficiary openly denied a request by her elder sister-in-law for the cash transfer [beneficiary 7, Kote]. In both these instances, it was unclear whether conflict was triggered by the cash transfer or whether it was a continuation of a pre-existing pattern of antagonism.

In extremely poor households, where food security was a matter of survival [beneficiary 4, Kote] or the family was so in debt they were at risk of eviction [beneficiary 3, Kote], a senior in-law received and spent the transfer on meat, fish or staples for the whole family instead of dairy or fruits for the pregnant woman. Beneficiary women in such households stated they shared out of duty, solidarity, charity and necessity.

Beneficiary woman: I had to give the cash transfer to my mother-in-law. Since we had nothing at home to eat, then it was my duty to give money to buy rice, salt, oil and other things for the kitchen … I had given her [the cash transfer] so that she could buy food for all of us in the family and I too had eaten what she bought.

[beneficiary 4, Kote]

Even so, such they often felt spontaneously required to justify not spending their cash transfer on fruits or dairy; for example, the above beneficiary woman exclaimed ‘*How can you stay alive eating exclusively apples and pomegranates?*’ [beneficiary 4, Kote]

## 5 Discussion

We showed how the context, implementation, and mechanism of a participatory women's group intervention created multiple, interlocking forms of social pressure on beneficiary women and their families to spend cash transfers on foods recommended by our NGO staff. Our observed mechanism may help explain how the combined women's group and cash transfer intervention achieved substantial improvements in dietary diversity, micronutrient adequacy, consumption of dairy foods, and relative intra-household allocation of dairy foods to pregnant women (Harris-Fry et al., 2018a), even if it may not have translated into impact on birthweight (Saville et al., 2018). In a context in which pregnant women are expected to eat least and last (Morrison et al., 2018), are perceived as having less need of nutrition than other household members (Morrison et al., 2018), and are found to have the lowest level of energy intake and micronutrient adequacy among adult household members (Harris-Fry et al., 2018b), improving their ability to spend cash on their own nutrition was a significant achievement.

The observed agreement between our qualitative findings – in which most respondents reported spending their cash on milk and fruit – and the quantitative findings of an independent dietary sub-study (Harris-Fry et al., 2018a) – in which micronutrient adequacy and consumption of dairy improved – suggests limited social desirability bias in our interview data. Our findings mirror pilot data from the trial surveillance system in early 2014, which found that 84 of 86 households reported spending their cash transfer on food for the pregnant women. Triangulating between the reports of mothers-in-law, elder sisters-in-law and beneficiaries from the same household did not reveal significant discrepancies between reported behaviours. Furthermore, most respondents, whether beneficiary woman, husband or mother-in-law, showed little inhibition in stating that the cash transfers were too small to make a difference to their lives and that future programmes should provide larger transfers. Nonetheless, we cannot eliminate the possibility that women tried to protect themselves from loss of standing with MIRA NGO when they asserted that they believed the cash transfers ought to be spent as instructed, even if such behaviour would in itself signal social pressure to appear compliant.

We believe the process through which women were encouraged to spend cash on their own nutrition most closely resembles the experience of a cash transfer programme in Lesotho (Pace et al., 2016, 2018) in which complementary labelling and messaging components spurred communities to police beneficiary spending of cash transfers against perceived waste. In our case, NGO staff were motivated to create and enforce spending recommendations for our cash transfers by fears that household members would fail to see their potential value for women's pregnancy and so confiscate them for their own purposes. NGO recommendations were not purely top-down driven but emerged out of an interactive process between group members, their facilitators and their supervisors which resulted in a group consensus. As group facilitators, their supervisors and community members enthusiastically promoted compliance, a social norm was created, which strongly compelled beneficiary women to spend their cash transfer on recommended foods, while considering purchases of ‘frivolous’ or ‘wasteful’ items, such as cosmetics or jewellery, socially unacceptable. This enabled most beneficiaries to spend cash on their own pregnancy, while preventing them from spending it on non-health purposes or on other household members' well-being. Moreover, households in extreme poverty and food insecurity still purchased and shared staple foods for the whole family.

Our initial hypothesis that women's groups might empower beneficiaries to exercise greater agency over cash transfers does not seem to have held. In fact, our conception of agency as rooted in non-interference from other household members seems to have been too narrow for a setting where constraints on women's use of cash came from community and NGO members in addition to household members. Under a broader definition of agency as non-interference from either household, community or NGO members, we might say our women's group intervention actually circumscribed beneficiary agency by constraining them to spend cash on health-related purposes.

Such constraints can be viewed as a form of ‘soft conditioning’ on transfer use (Pace et al., 2016, 2018; Skovdal et al., 2013). ‘Hard’ conditions refer to formal requirements for continued receipt of cash transfers which are enforced by an external implementing agent. ‘Soft’ conditions refer to implicit constraints on transfer use that arise from community members' own interpretation of programme guidelines. No formal requirements existed for beneficiaries in our programme to spend cash transfers on their own pregnancy, but community members and local women's group facilitators sometimes took it upon themselves to enforce this anyway.

A theory of how the context, implementation and mechanism of our participatory women's group intervention circumscribed beneficiary agency over cash transfers should account for three phenomena: concertive control, imperfect implementation and labelling. ‘Concertive control’ (Barker, 1993) refers to strategies of collective control whereby groups invent and enforce own rules of behaviour based on their interpretations of an evolving value consensus. In a notable ethnography of ‘participatory democracy’ at the workplace in the United States, Barker (1993) observed how newly created, self-managed work teams gradually imposed ever-more formalistic rules of reward and punishment on themselves to satisfy their value consensus until they had effectively replaced their former top-down bureaucracy with a bottom-up bureaucracy of their own making.

Despite clear differences in context, this process may serve as a model for our study, as we similarly observed group facilitators, group members and community members creating and enforcing their own rules of transfer use based on their interpretations of what these transfers were for. Of particular resonance is Barker's (1993) observation that concertive control can be more powerful and pervasive than top-down management, because group members are always around to watch each other, whereas managers can only monitor a few at a time. In our case, beneficiary women were put under the ‘eye of the norm’ (Barker, 1993) through the efforts of NGO and community members to monitor their usage of cash. This limited their ability to spend cash on non-health purposes but enhanced their ability to spend it on their own pregnancy.

Imperfect implementation limited the bottom-up nature of our intervention in some areas, as one group supervisor threatened to withdraw cash transfers, if women in the community did not follow their recommendations, and another promised universal instantiation of the programme if they did so. No such contingencies existed in our intervention. Gaarder (2012) commented on the invariable risk of imperfect implementation in real-world unconditional cash transfer programmes and asked whether field staff on the ground ‘know and understand the implication of unconditionality [or] agree and communicate it in that spirit’. Qualitative studies of cash transfer programmes in Peru, Indonesia and Nicaragua have found programme officers ‘empowering themselves’ to collect receipts from beneficiaries with the warning that the transfers would stop if they wasted the money, even though no such legal condition existed (Jones et al., 2007; Syukri et al., 2010; Adato and Roopnaraine, 2010).

In our trial, imperfect implementation may have created confusion around cash transfer policy. Even if few beneficiaries explicitly stated that the cash transfer was conditional in our interviews, our NGO staff thought this belief might have arisen. Previous studies in Malawi, Ecuador, Morocco and Zimbabwe have found 27–70% of households receiving unconditional cash transfers believing they were in fact conditional (Benhassine et al., 2015; Robertson et al., 2013; Schady and Araujo, 2008; Kilburn et al., 2017). Indeed, a few respondents in our sample stated a belief that our NGO could find out if women had eaten the food they were supposed to eat by measuring the weight of the baby that was eventually born. If respondents believed their community could stop receiving cash transfers in this way, they may have felt forced to shift spending in accordance with perceived instructions.

Direct messages from facilitators in group meetings concerning the purpose of the cash transfer interacted with concertive control and some instances of imperfect implementation to alter the ‘social meaning’ (Zelizer, 1989) of cash transfers. Beneficiary women, household members, and NGO staff universally affirmed that beneficiaries ought to spend their transfer on food, because they were given it to spend on food, and strongly condemned spending on non-food purposes. Particularly striking is the analogy drawn by one husband between household spending of cash transfers and bureaucrat spending of government funds (Section 4.3.1) given the fact that labelling effects were first discovered in the context of local government spending (Courant et al., 1978). As a result, household members and beneficiary women may have felt a strong obligation to spend cash on NGO-approved purposes and enforce such spending within their own household. Indeed, the transfers seem to have become so strongly associated with NGO compliance in the minds of some respondents that they seemed confused about the very idea that beneficiaries could spend cash transfers in their own way.

The extent to which the observed lack of agency expansion should concern policy-makers depends on their assumptions, theories and values. First, soft conditions on cash transfers may be inefficient if beneficiaries are better informed about optimal use of cash than social planners. For example, in our study, group facilitators' budgetary recommendations were strictly not evidence-based but emerged out of a consensus attempt at collectively making the best use of available resources. There is no evidence that drinking half a litre of milk a day improves birthweight, only broad evidence that dietary diversity and micronutrient adequacy is important (Bhutta et al., 2013). Nonetheless, group facilitators and members were arguably better informed about ways to enhance nutrition and health as a collective than individual beneficiaries and their budgetary recommendations and guidelines might have helped translate vague notions of optimal diet into concrete action.

Second, soft conditions may be considered paternalistic from a human rights perspective, particularly if the purpose of programmes is to mitigate the effects of extreme poverty. However, only classic liberal theorists define freedom as the absence of external interference (Berlin, 1959). Republican theorists (Pettit, 1997) define freedom as the presence of legitimate institutions that prevent arbitrary interference; thus, collectively self-imposed rules are exactly the type of situation that enable freedom. Indeed, republicans may argue behavioural constraints designed by the NGO and the community to promote pregnant women's health and nutrition carry greater legitimacy than arbitrary confiscation of cash transfers by other household members. More broadly, collectively agreed constraints on individual behaviour are often necessary to reach collective goals and produce public goods (Gram et al., 2018b, Gram et al., 2018c). Our findings indicate a need for considering conceptions of agency, which distinguish between legitimate and illegitimate interference (Gram et al., 2017).

Future research may continue to explore the complex ethical, social, and behavioural implications of combining participatory women's groups with cash transfers. Previous quantitative and qualitative studies have framed women's agency over cash transfers primarily as an issue of intra-household gender inequality (Adato and Roopnaraine, 2010; Yildirim et al., 2014; Slater and Mphale, 2008; Scott et al., 2017). With few exceptions (Forde et al., 2011), hard and soft conditions on beneficiary spending are rarely considered manifestations of power. Future work might analyse such power relations through an intersectional lens which combines insights from both gender, community and NGO realms. Understanding such intersections may be key to determining whether policy-makers should necessarily think of our observed soft conditions as problematic or simply emblematic of the ‘necessary contradictions’ (Cornish and Ghosh, 2007) inherent in combining participatory women's groups with cash transfers.
